# Data on the diversity of lactic acid bacteria isolated from raw and fermented camel milk

**DOI:** 10.1016/j.dib.2020.105956

**Published:** 2020-07-03

**Authors:** Elvira Nagyzbekkyzy, Dinara Sembayeva, Ainur Sarsenova, Nurlan Mansurov, Altyn Moldabayeva, Nazira Moldagulova

**Affiliations:** a Institution International Academy of Ecology, Ryskulbekova, 16 VP, 010000, Nur-Sultan, Kazakhstan; b Limited Liability Partnership “Ecostandart.kz” (LLP “Ecological standard.kz”), Nur-Sultan, Kazakhstan; c Nazarbayev University, Nur-Sultan, Kazakhstan; d Non-commercial joint-stock company “Astana Medical University”, Kazakhstan

**Keywords:** Lactic acid bacteria, Camel milky, Fermented camel milk, Diversity

## Abstract

Lactic acid bacteria (LAB) are the dominant and advantageous microorganisms of raw and fermented camel milk; these bacteria have the potential to develop functional camel-milk-derived products and can be used in dairy technology. This article presents data on the diversity of LAB, isolated from raw and fermented camel milk. In total, from two samples of raw camel milk and one sample of fermented camel milk, seventeen isolates of LAB were isolated. The data of genetic identification of strains, which was performed through analysis of the 16S rRNA gene sequence, is presented. According to this data, the prevailing number of LAB belong to the *Lactobacillus* genus – 53%. Following species of Lactobacillus bacteria were determined – *fermentum, casei, curizae, oryzae, brevis, plantarum, rhamnosus, paracasei.* The next prevailing number of lactic acid bacteria belong to the *Pediococcus* genus – 23%, represented by *acidilactici and pentosaceus* species. Lactic acid bacteria of *Weissella* and *Enterococcus* genera comprised 12% each from total abundance. These results can be used for a further selection of potential starter cultures for functional camel-milk-derived products.

Specifications table

**Table:** 

Subject	Applied Microbiology and Biotechnology
Specific subject area	Isolation and identification of lactic acid bacteria, isolated from raw and fermented camel milk with future potential in the development of starter cultures
Type of data	Tables and figure
How data were acquired	Microscope MS 300X (MICROS, St. Veit an der Glan, Austria), 3730xl DNA Analyzer (Applied Biosystems, Foster, USA), SeqScape 2.6.0 software (Applied Biosystems, Foster, USA).
Data format	Raw and analyzed data.
Parameters for data collection	Data were collected by seeding raw and fermented camel milk samples on plates with MRS agar.
Description of data collection	The data were collected by conducting microbiological seeding of raw and fermented camel milk samples on nutrient media, followed by isolation LAM and identification.
Data source location	Nur-Sultan, Kazakhstan
Data accessibility	Data is included in this article. The sequences of the fragments of the 16S r RNA gene are deposited in the NCBI database, under accession numbers MT598199.1, MT598200.1, MT598201.1, MT598202.1, MT598203.1, MT598204.1, MT598205.1, MT598206.1, MT598207.1, MT598208.1, MT598209.1, MT598210.1, MT598211.1, MT598212.1, MT598213.1, MT598214.1, MT598215.1. (https://www.ncbi.nlm.nih.gov/nuccore/)

## Value of the Data

• The right starter culture plays a crucial role in the success of the fermented milk product. This data would be valuable for further studies to select beneficial LAB with unique biotechnological properties.
• The value of the data is on the possibility of finding the novel species of LAB among the LAB, isolated from raw and fermented camel milk.
• This data can be an impetus for the development of a new domestic starter for camel milk.
• Researchers who are engaged in studying of new strains of the LAB could use this data.

## 1 Data

Camel milk and fermented camel milk are popular drinks among the population of Kazakhstan. Due to the unique therapeutic potential and absence β-LG, camel milk has a focus area of research in health science and nutrition [1]. Fermented camel milk, named shubat, is also known for its medicinal and dietary properties [2]. LAB is the dominant population in raw and fermented milk; they produce various antimicrobials such as organic acids and hydrogen peroxide, antifungal peptides and bacteriocins and play a crucial role in food fermentation processes [3]. The isolation and characterization of resident LAB in raw and fermented camel milk are essential for further development starters of functional camel-milk-derived products.

**Fig. 1:**
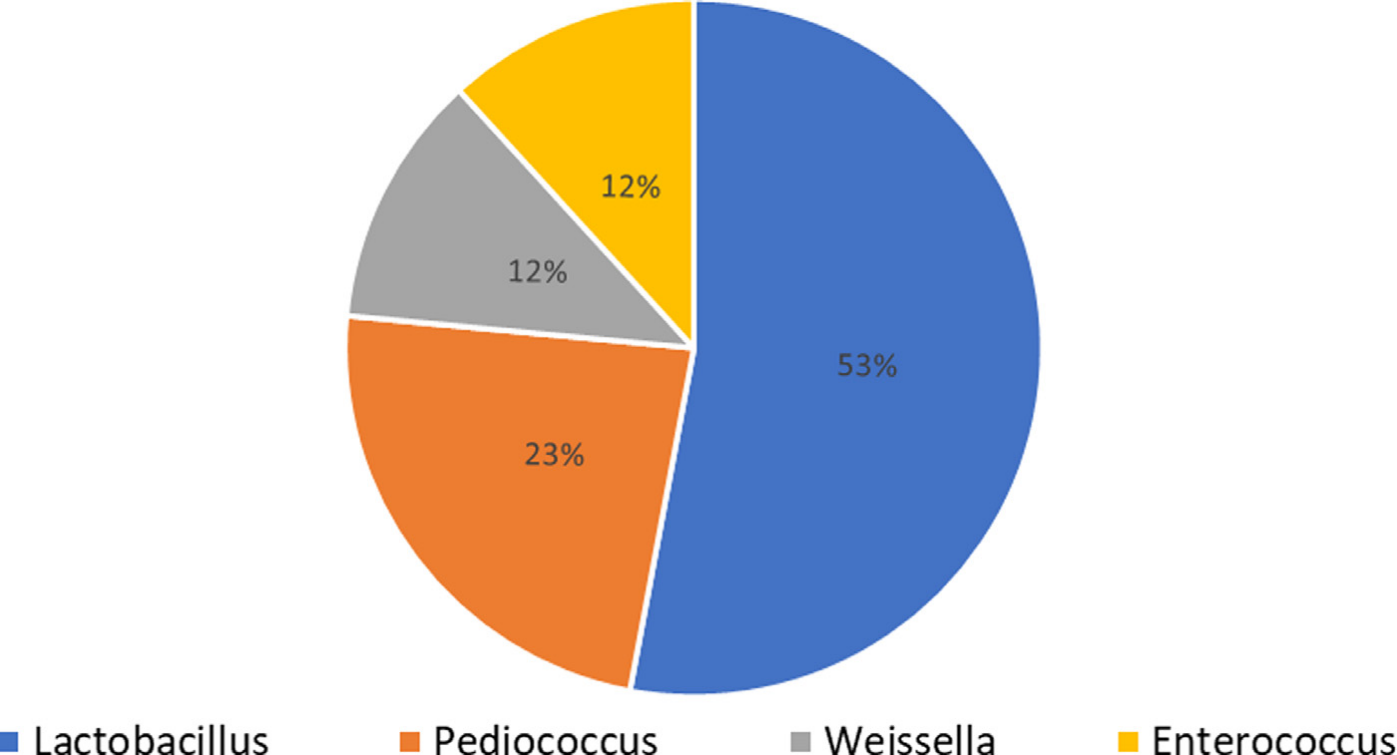
The diversity of lactic acid microorganism's genus, based on 16S rRNA identification.

The data of this article provides data on the isolation of LAB from raw and fermented camel milk and their identification. In total, three samples were used for data collection (two samples of raw camel milk and 1 sample of shubat – traditional fermented camel milk in Kazakhstan). From two samples of camel milk, eight LAB were isolated, from fermented camel milk, nine LAB were isolated. Table 1 presents the data of microscopic characteristics of isolates. By molecular identification based on the analysis of the 16S rRNA gene's partial sequence, the isolates were assigned to the genus *Lactobacillus, Pediococcus, Weissella*, and *Enterococcus*. Table 2 presents the nucleotide sequences of isolate's 16S rRNA gene and their similarity to available nucleotide sequences deposited in the Gene Bank databases. For three strains of lactic acid bacteria L-003, L-006, L-015, the percent of identity to their affiliations were 89%, 93%, 95%, respectively. It could indicate that novel species of LAB possibly were isolated from raw and fermented camel milk. On Fig. 1 presented the diversity of LAB, isolated from raw and fermented camel milk.

**Table 1:** Microscopical characteristics of isolates.

Isolate	Sample	Place of sample isolation	Microscopical characterization	
			Gram stain	Cell morphology
L-001 L-002 L-011 L-008 L-013	camel milk	Turkestan region	positive positive positive positive positive	rod cocci cocci oval rods rod
L-003 L-004 L-005 L-006 L-007 L-009 L-010 L-014 L-015	fermented camel milk	Turkestan region	positive positive positive positive positive positive positive positive positive	rod rod cocci rod rod cocci cocci cocci small cocci
L-017 L-018 L-019	camel milk	Kyzylorda region	positive positive positive	rod rod rod

**Table 2:** Genetic identification of strains based on partial sequence of 16S rRNA.

Isolate	Accession number in GenBank	Accession number to an identical sequence in GenBank	Species affiliation	Percent identity (%)
L-001	MT598199.1	MH532286.1	*Lactobacillus fermentum*	100
L-002	MT598200.1	MF179633.1	*Pediococcus acidilactici*	*100*
L-011	MT598201.1	MH473167.1	*Pediococcus pentosaceus*	99
L-008	MT598202.1	MH398517.1	*Weissella confusa*	99
L-013	MT598203.1	MK418664.1	*Lactobacillus plantarum*	100
L-003	MT598204.1	MF405178.1	*Lactobacillus curieae*	89
L-004	MT598205.1	NR041893.1	*Lactobacillus casei*	100
L-005	MT598206.1	GU983698.1	*Enterococcus lactis*	100
L-006	MT598207.1	MG462090.1	*Lactobacillus oryzae*	93
L-007	MT598208.1	MH817736.1	*Lactobacillus paracasei*	100
L-009	MT598209.1	FR873980.1	*Pediococcus pentosaceus*	97
L-010	MT598210.1	KY466904.1	*Pediococcus acidilactici*	98
L-014	MT598211.1	MH346266.1	*Enterococcus faecium*	99
L-015	MT598212.1	MH398517.1	*Weissella confusa*	95
L-017	MT598213.1	MK418585.1	*Lactobacillus rhamnosus*	98
L-018	MT598214.1	MK408481.1	*Lactobacillus brevis*	*100*
L-019	MT598215.1	MK418666.1	*Lactobacillus plantarum*	99

## 2 Experimental design, materials, and methods

### 2.1 Source for isolation LAB

For data producing, two samples of raw camel milk were collected: from Social Entrepreneurship Corporation «Zhelmaya» in Turkestan region and private compound in the Kyzylorda region. Also, a sample of fermented camel milk – shubat, collected from Social Entrepreneurship Corporation «Zhelmaya», was used for data-producing. The samples of fresh raw and fermented camel milk were collected to the sterile glass tubes and delivered to the laboratory at 4 °C.

### 2.2 Isolation of LAB cultures

One milliliter from each sample was transferred to the tubes with 7 mL of MRS broth (HiMedia Lab Pvt., Mumbai, India) and inoculated at 37 °C under microaerophilic conditions. After 24 h of incubation 0.1 mL of suspension spread-plated onto MRS agar (HiMedia Lab Pvt., Mumbai, India) to isolate the LAB. The plates were incubated at 37 °C for three days. Colonies with morphological differences in color, shape, and size were picked and purified by streaking. Pure cells of isolates were maintained at −20 °C in the culture broth, which was supplemented with 10% glycerol, also the cultures stored in a hermetically sealed form in the refrigerator at + 4–8 °C.

### 2.3 Microscopical characterization

Smears of the LAB strains were prepared on a clean glass slide. The air-dried smears were fixed by heating. The fixed smears were washed with water after flooding with crystal violet. Then were flooded with mordant Gram's iodine, decolorized with ethanol, washed with water, and counterstained with safranin for 45 s. Then the glass slides were washed with water, dried with tissue paper and examined under oil immersion (100 ×) [4].

### 2.4 Molecular identification of bacterial isolates

Genomic DNA was isolated from pure LAB cultures using Wizard Genomic DNA Purification Kit A11125 (Promega Corporation, Madison, USA) following the manufacturer's protocol. The genotyping was performed using amplification of the fragment of the 16S rRNA gene according to the following protocol by Shevtsov A. B. et al. [5]. The PCR reaction was performed with universal primers – (5′-agagtttgatcctggctcag-3′) and (5′-ggactaccagggtatctaat-3′) in a total volume of 30 μl. Purification of PCR products from unbound primers was performed using Exonuclease I enzymes (Fermentas, Vilnius, Lithuania) and alkaline phosphatase (Fermentas, Vilnius, Lithuania). The sequencing reaction was carried out using the BigDye® Terminator v3.1 Cycle Sequencing Kit (Applied Biosystems, Foster, USA) according to the manufacturer's instructions, followed by fragment separation on a 3730xl DNA Analyzer (Applied Biosystems, Foster, USA). The nucleotide sequences of the 16S rRNA gene of LAB cultures were analyzed and aligned into a common sequence in SeqScape 2.6.0 software (Applied Biosystems, Foster, USA). After that, terminal fragments (nucleotide sequences of primers with fragments having a low-quality index) were removed, which allowed us to obtain a nucleotide sequence of more than 650 bp in length. The later was identified in GeneBank using the BLAST algorithm.

## Declaration of Competing Interest

The authors declare that they have no known competing financial interests or personal relationships which have or could be perceived to have, influenced the work reported in this article.

## Appendix Supplementary materials

Image, application 1
